# Correlation between Macular Neovascularization (MNV) Type and Druse Type in Neovascular Age-Related Macular Degeneration (AMD) Based on the CONAN Classification

**DOI:** 10.3390/biomedicines10102370

**Published:** 2022-09-22

**Authors:** Daniel Rudolf Muth, Mario Damiano Toro, Anahita Bajka, Kamil Jonak, Roman Rieder, Myrtha Magdalena Kohler, Jeanne Martine Gunzinger, Eric H. Souied, Michael Engelbert, K. Bailey Freund, Sandrine Anne Zweifel

**Affiliations:** 1 Department of Ophthalmology, University Hospital Zurich (USZ), University of Zurich (UZH), 8091 Zurich, Switzerland; 2 University of Zurich (UZH), 8006 Zurich, Switzerland; 3 Department of General and Pediatric Ophthalmology, Medical University of Lublin, 20-093 Lublin, Poland; 4 Eye Clinic, Public Health Department, University of Naples Federico II, 80138 Naples, Italy; 5 Department of Biomedical Engineering, Lublin University of Technology, 20-618 Lublin, Poland; 6 Department of Psychiatry, Psychotherapy and Early Intervention, Medical University of Lublin, 20-093 Lublin, Poland; 7 Department of Ophthalmology, Centre Hospitalier Intercommunal de Creteil, University Paris Est Creteil, 94000 Creteil, France; 8 Vitreous Retina Macula Consultants of New York, New York, NY 10022, USA; 9 Department of Ophthalmology, NYU Grossman School of Medicine, New York, NY 10016, USA

**Keywords:** subretinal drusenoid deposits, SDDs, reticular pseudodrusen, spectral domain optical coherence tomography, OCT, MNV, macular neovascularization, CNV, age-related macular degeneration, AMD

## Abstract

To investigate associations and predictive factors between macular neovascularization (MNV) lesion variants and drusen types in patients with treatment-naïve neovascular age-related macular degeneration (AMD). Methods: Multimodal imaging was retrospectively reviewed for druse type (soft drusen, subretinal drusenoid deposits (SDDs) or mixed) and MNV type (MNV 1, MNV 2, MNV 1/2 or MNV 3). The Consensus on Neovascular AMD Nomenclature (CONAN) classification was used for characterizing MNV at baseline. Results: One eye of each eligible patient was included (*n* = 191). Patients with predominant SDDs had an increased adjusted odds ratio (aOR) for MNV 2 (23.4453, *p* = 0.0025) and any type of MNV 3 (8.7374, *p* < 0.0001). Patients with MNV 1/2 had an aOR for predominant SDDs (0.3284, *p* = 0.0084). Patients with MNV1 showed an aOR for SDDs (0.0357, *p* < 0.0001). Eyes with SDDs only without other drusen types showed an aOR for MNV 2 (9.2945, *p* < 0.0001). Conclusions: SDDs represent a common phenotypic characteristic in AMD eyes with treatment-naïve MNV. The aOR for eyes with predominant SDDs to develop MNV 2 and MNV 3 was much higher, possibly due to their location in the subretinal space. The predominant druse type may help to predict which type of MNV will develop during the course of AMD.

## 1. Introduction

With the availability of high-resolution retinal imaging, the classification of macular neovascularization (MNV) and drusen has evolved. Based on anatomical localization and multimodal imaging, including fluorescein angiography and spectral domain optical coherence tomography (SD-OCT), a revised classification scheme was proposed by Freund et al. [1]. This classification system built upon Grossniklaus’ and Gass’ original observations from histopathologic slides of neovascular age-related macular degeneration (AMD) differentiating between vessels confined to the sub-retinal pigment epithelium space, described as type 1 MNV, and vessels proliferating above the retinal pigment epithelium in the subneurosensory, subretinal space, described as type 2 MNV [2]. Gass had recognized that a distinction between type 1 and type 2 MNV based on biomicroscopic and fluorescein angiographic findings “is not always easy and in some cases impossible” [3]. Incorporating findings from fluorescein angiography and OCT for grading neovascular AMD, higher incidences of intraretinal neovascularization, described as type 3 MNV, and mixed types 1 and 2 were found compared with those reported in prior studies using fluorescein alone [4]. A new nomenclature was proposed by the Consensus on Neovascular Age-Related Macular Degeneration Nomenclature (CONAN) Study Group in 2020 [5].

Optical coherence tomography has improved our understanding of not only MNV types, but has also expanded and refined distinctions between drusen types. The clinical appearance of reticular pseudodrusen, first described in 1990 [6], could be linked to aggregations containing typical drusen-associated material located in the subretinal space, which were termed subretinal drusenoid deposits [7]. Subretinal drusenoid deposits have a high prevalence in AMD, which has been underestimated prior to the SD-OCT era [7]. Subretinal drusenoid deposits have been recognized as an additional feature of early and both forms of late AMD, called geographic atrophy/complete retinal pigment epithelium and outer retinal (cRORA) and neovascular AMD [8,9,10]. Late AMD is associated with vision loss [11]. The latest report of the Age-Related Eye Diseases Study 2 concluded that subretinal drusenoid deposits significantly contribute to the development of late AMD stages, especially in patients who already present subretinal drusenoid deposits at an early, low-disease-severity stage [11]. Subretinal drusenoid deposits mainly seemed to contribute to the development of geographic atrophy [11]. However, soft drusen and subretinal drusenoid deposits were also found to be risk factors for MNV [12]. There are published data suggesting that patients with subretinal drusenoid deposits are more likely to develop macular type 3 MNV [13,14].

The purpose of this study was to assess the predictive odds ratios between drusen type and MNV lesion variants in patients with treatment-naïve neovascular AMD [5].

## 2. Materials and Methods

### 2.1. Ethics 

Ethics Committee approval was obtained from the Local Ethics Committee of the Canton of Zurich (approval number: PB_2016-00264). This study adheres to the tenets of the 1964 Declaration of Helsinki and its later amendments.

### 2.2. Study Design

This is a single-center, retrospective, observational study conducted at the Department of Ophthalmology of the University Hospital of Zurich (USZ), Switzerland.

### 2.3. Data Collection

All fluoresceine angiographies (FA) performed between 2011 and 2013 at the University Hospital of Zurich were screened for the presence of treatment-naïve macular neovascularization (MNV). Inclusion criteria for this study were the presence of treatment-naïve MNV secondary to AMD as evidenced by multimodal imaging, including FA, spectral-domain optical coherence tomography (SD-OCT), fundus autofluorescence (FAF) imaging and by clinical examination in patients aged ≥50 years. All neovascular lesion types, including polypoidal choroidal vasculopathy (PCV), were evaluated. Eyes with PCV were included only when structural signs of AMD were also present. Eyes with central serous chorioretinopathy (CSC) with or without PCV were excluded. In patients with no available baseline images of the study eye prior to the development of MNV, druse type was evaluated in the fellow eye provided there was no evidence of MNV. Only one eye per participant was selected and included in the study. If both eyes of the same patient were eligible, one eye was chosen randomly.

Exclusion criteria included patients with bilateral MNV at baseline, eyes with predominantly fibrotic lesions and those with such poor image quality that reliable classification of MNV and drusen was not possible.

Patients with bilateral MNV at baseline were excluded from the study as there would not be any pre-conversion images or a non-neovascular fellow eye to allow the grading of drusen type. 

Neovascularizations that were confined to the space under the retinal pigment epithelium (RPE) were defined as MNV 1. PCV was considered an aneurysmal variant of MNV 1 and therefore subsumed under MNV 1. Neovascularizations proliferating above the RPE, in the subneurosensory, subretinal space, were defined as MNV 2. Cases with mixed type 1 and 2 lesions were recorded as a separate subgroup termed MNV 1/2. Cases with multiple lesion types including any type 3 lesion (MNV 1/3 and MNV 2/3) were summarized under MNV 3.

Descriptive statistics regarding the participant demographics are listed in Table 1.

FA images were obtained with either a fundus camera system (Carl Zeiss AG, Oberkochen, Germany) or the Heidelberg Viewing Module (version 6.0.9.0) included in our Spectralis SD-OCT device (version 1.9.10.0; Heidelberg Engineering GmbH, Heidelberg, Germany). Indocyanine green angiography (ICGA) and FAF images were obtained from all patients using the confocal scanning laser ophthalmoscope (CSLO) (Heidelberg Retina Angiograph, HRA2, Heidelberg Engineering, Heidelberg, Germany). 

### 2.4. Image Grading and Analysis

**Figure 1:**
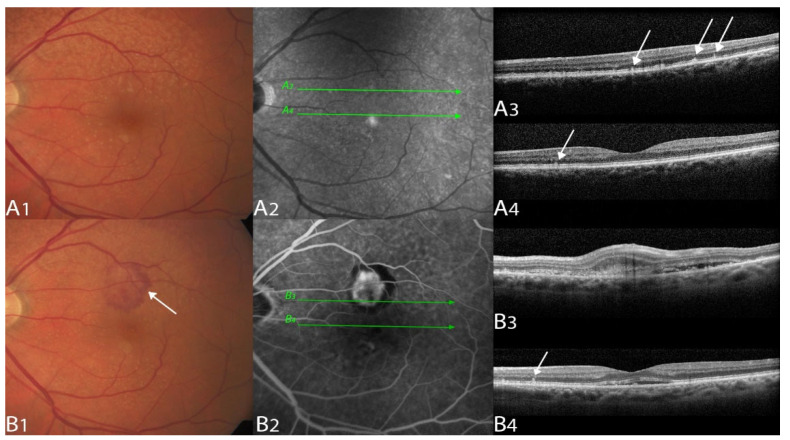
Multimodal imaging example of a type 2 macular neovascularization.

**Figure 2:**
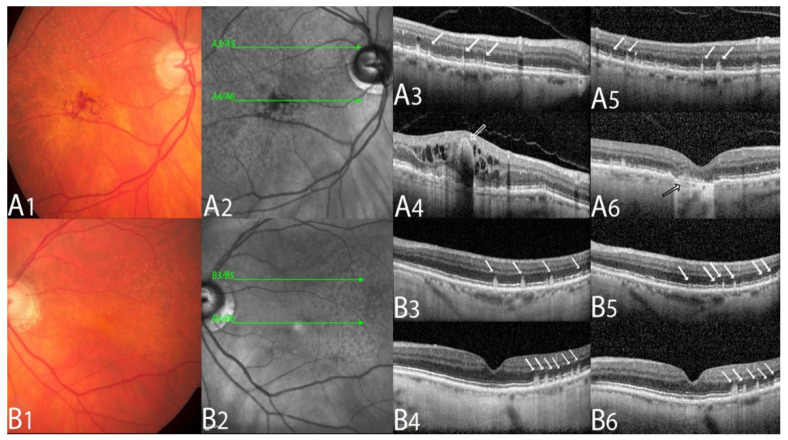
Multimodal imaging example of a type 3 macular neovascularization.

Neovascular lesions were subtyped according to CONAN criteria [1,5]. See Figure 1 and Figure 2 for examples [1,4].

Drusen types were categorized into soft drusen and SDDs. SDDs were considered present when there was OCT evidence of ≥5 definite SDDs above the RPE in >1 B-scan, with consistent changes in either the near-infrared imaging or the blue light channel, as previously described [7]. Soft drusen were determined from color fundus photographs and confirmed by SD-OCT. If both types of drusen were present, the predominance of either soft drusen or SDDs was determined by two experienced graders; in the case of discordance among the readers, a senior retina specialist was consulted (SAZ) (see Table 2).

The druse type that occupied the greater number of subfields in the Early Treatment Diabetic Retinopathy Study (ETDRS) macular grid was determined as the predominant druse type.

### 2.5. Statistical Analysis

Data were organized in Microsoft Excel (Microsoft Corp., Redmond, WA, USA) and statistically analyzed using SPSS software version 23 (SPSS Inc., Chicago, IL, USA) and R.app v4.1.0 GUI 1.76 for MacOS (The R Foundation for Statistical Computing c/o Institute for Statistics and Mathematics, 1020 Vienna, Austria). Descriptive statistics, such as the mean and standard deviation (SD), were computed. The number of neovascular AMD lesions as identified by the anatomic classification system was recorded. 

Adjusted odds ratios (aORs), as a predictor between druse type and MNV type, were calculated in R using binary logistic regression. As the characteristic structural changes in AMD are age-related, age will not be normally distributed in our dataset. The statistical feature “age” will additionally contribute to the odds ratios (ORs). As we wanted to evaluate the effect of drusen on MNV development alone, we adjusted the ORs for age. The statistical significance level (α) was defined as 0.05. Statistical analysis results with a *p*-value less than 0.05 were interpreted as statistically significant. 

## 3. Results

A total of 3980 eyes with treatment-naïve neovascular AMD were screened. Among these, 536 eyes met the eligibility criteria. Of those, 154 eyes were excluded due to advanced fibrovascular scarring and/or poor image quality precluding a reliable classification of MNV and drusen type. Eventually, 382 eyes of 191 patients were eligible. Only 1 eye per patient was included, resulting in 191 study eyes (*n* = 191) (Table 1). If both eyes were eligible, one eye was chosen randomly. Patients with the predominant druse type of SDD had higher aORs for MNV 2 and MNV 3 (Table 3). Patients with MNV 1 were less likely to have predominant SDDs (Table 3). No clear predictor for any druse type was found for mixed MNV 1/2 lesions. Patients with soft drusen showed the highest overall aOR with MNV 1 lesion (Table 3).

History of diabetes, arterial hypertension and glaucoma did not show statistically significant correlations (Phi/Cramer-v) with neither MNV type nor druse type (Table 4). 

## 4. Discussion

The subretinal space is an unusual location for the extracellular deposition of material, and not only changes in the sub-RPE location, but also changes anterior to the RPE cells, may play an important role in AMD pathogenesis. The presence of SDDs seems to be associated with thin choroidal thickness [15,16]. Eyes with sub-RPE predominant soft drusen show the highest overall odds ratio for developing neovascular AMD [17]. We observed the same trend in our data (Table 3). However, subretinal SDDs clearly seem to be associated with late AMD, including complete outer retinal atrophy (cRORA) as well as MNV in AMD [11,18,19,20]. There seemed to be debate as to whether SDDs could be linked to an AMD MNV subtype [21]. Cohen et al. were the first to observe a correlation between SDD and the MNV 3 subtype [13]. Marsiglia et al. demonstrated that patients with MNV 1 were less likely to have SDDs and that patients with MNV 3 were more likely to have SDDs in their non-neovascular fellow eye [14]. They did not observe an association between SDDs and MNV 2, which might be due to the low number of only 10 eyes with MNV 2 in their series [14]. Applying the CONAN Study Group criteria for MNV classification to this dataset, we could demonstrate that, in AMD patients with treatment-naïve MNV, SDDs were more likely to be a risk factor for MNV 2 and MNV 3 as compared with soft drusen (Table 3). MNV 2 occurs in the subretinal space above the RPE. MNV 3 originates intraretinally (supra-RPE) and penetrates the RPE only in later stages. Therefore, SDDs seem to be a plausible risk factor for the development of both the MNV 2 and MNV 3 subtypes. This association is supported by findings from Spaide et al., Rabiolo et al. and Lee et al., who demonstrated that SDDs would preferably progress to MNV 2 and MNV 3 [16,19,20,22]. We could confirm this finding with MNV 2 and with MNV 3 for predominant SDDs. Interestingly, we did not find a statistically significant odds ratio for SDDs only with any type of MNV 3. This might be due to our classification of the presence of any MNV 3 under one group. Hence, the MNV 3 group in our series also included mixed lesions, such as MNV 1/3 and MNV 2/3, making it difficult to find a statistical predictor. 

Ahmed et al. frequently discovered MNV 2 without the presence of extracellular deposits [23]. This highlights that SDDs may be difficult to reliably detect. Rabiolo et al. also recommended using multimodal imaging with at least two different modalities to reliably detect SDDs [22]. 

The limitations of this study include its retrospective nature and the fact that graders were not masked to the original diagnosis of neovascular AMD. The non-neovascular fellow eye was used to identify the type of drusen in patients with no available baseline images of the study eye prior to the development of MNV; however, this is supported by prior studies showing intraindividual symmetry in eyes with AMD [24,25,26,27].

## 5. Conclusions

The anatomical classification of MNV, which incorporates FA and SD-OCT findings, seems to provide more accurate information about associations between the type of drusen and type of MNV. Predicting the type of MNV for patients with intermediate AMD might inform more personalized patient care with respect to monitoring and future treatment.

## Figures and Tables

**Table 1:** Demographic data.

Total patients screened (n_screen_)	1990 (3980 eyes)
Patients identified as suitable (n_patient_)	268 (536 eyes)
Patients removed due to fibrovascular scarring or poor image quality (n_dropped_)	77 (154 eyes) (29%)
Patients included (n_patient_)	191 (382 eyes) (71%)
Eyes included (1 per patient) (*n*)	191 eyes
Localization OD/OS	97 (51%)/94 (49%)
Gender female/male	117 (61%)/74 (39%)
Age mean (years) ± SD	77.9 ± 7.8
BCVA mean ± SD	56.6 ± 21.2 ETDRS letters
Glaucoma	22 (12%)
Arterial hypertension	92 (48%)
Diabetes mellitus	17 (9%)

Legend: OD, oculus dexter; OS, oculus sinister; SD, standard deviation of the arithmetic mean; BCVA, best-corrected visual acuity, ETDRS, Early Treatment Diabetic Retinopathy Study.

**Table 2:** Descriptive statistics of drusen types and MNV types.

	Total	MNV 1	MNV 2	MNV 3	MNV Mixed 1/2
Eyes included (*n*)	191 (100%)	80 (42%)	23 (12%)	49 (26%)	39 (20%)
Soft drusen only	38 (20%)	34 (43%)	0 (0%)	1 (2%)	3 (8%)
Predominant soft drusen	43 (23%)	32 (40%)	1 (4%)	4 (8%)	6 (15%)
SDD only	39 (20%)	3 (4%)	14 (61%)	13 (27%)	9 (23%)
Predominant SDD	71 (37%)	11 (14%)	8 (35%)	31 (63%)	21 (54%)

Legend: *n*, number of included eyes; SDD, subretinal drusenoid deposit; MNV, macular neovascularization.

**Table 3:** Predictors (aOR adjusted for age) between druse and MNV type.

	aOR	95% CI	*p*
**MNV 1**			
Soft drusen only	19.0008	7.0019; 66.9073	**<0.0001**
Predominant soft drusen	27.9873	13.0180; 64.5909	**<0.0001**
SDD only	0.0806	0.01867; 0.2395	**<0.0001**
Predominant SDD	0.0357	0.0155; 0.0768	**<0.0001**
**MNV 2**			
Soft drusen only	n.s.	n.s.	0.8700
Predominant soft drusen	0.0427	0.0023; 0.2165	**0.0025**
SDD only	9.2945	3.6536; 24.9841	**<0.0001**
Predominant SDD	23.4453	4.6190; 429.4434	**0.0025**
**MNV 3**			
Soft drusen only	0.0661	0.0037; 0.3243	**0.00853**
Predominant soft drusen	0.1145	0.0376; 0.2849	**<0.0001**
SDD only	n.s.	n.s.	0.3198
Predominant SDD	8.7374	0.5105; 26.5916	**<0.0001**
**MNV 1/2 mixed**			
Soft drusen only	0.2816	0.0650; 0.8484	**0.0455**
Predominant soft drusen	0.3284	0.1364; 0.7268	**0.0084**
SDD only	n.s.	n.s.	0.6690
Predominant SDD	0.3284	0.1364; 0.7268	**0.0084**

Legend: 95% CI, 95% confidence interval; aOR, adjusted odds ratio; MNV, macular neovascularization; NA, not applicable; n.s., not statistically significant; *p*, probability of binary logistic regression (defined significant when *p* < 0.05; SDD, subretinal drusenoid deposit.

**Table 4:** Correlations (Phi/Cramer-V) between MNV type and druse type with systemic factors.

	Coeff.	95% CI	*p*
MNV type vs. Glaucoma	0.0757	0.0000; 0.0966	0.9063
MNV type vs. Hypertension	0.1153	0.0000; 0.1677	0.5471
MNV type vs. Diabetes	0.0726	0.0000; 0.0887	0.9221
Druse type vs. Glaucoma	0.0788	0.0000; 0.1048	0.8828
Druse type vs. Hypertension	0.1091	0.0000; 0.1321	0.6556
Druse type vs. Diabetes	0.1269	0.0000; 0.1591	0.4171

Legend: 95% CI, 95% confidence interval; Coeff., correlation coefficient of Phi/Cramer-V correlation for categorial/nominal data; MNV, macular neovascularization; n.s., not statistically significant; *p*, Pearson’s Chi-squared *p*-value (defined as significant when *p* < 0.05); SDD, subretinal drusenoid deposit.

## Data Availability

The data, aside from the data published in this manuscript, are not publicly available due to privacy restrictions.
